# Supplementary material for: IFT88 maintains sensory function by localising signalling proteins along Drosophila cilia
Source: Life Sci Alliance. 2024 Feb 19;7(5):e202302289. doi: 10.26508/lsa.202302289 (PMC10876440; doi:10.26508/lsa.202302289)
Supplement: Supplementary file 4 [file LSA-2023-02289_SdataF3.2.pdf]

Anti-GFP:

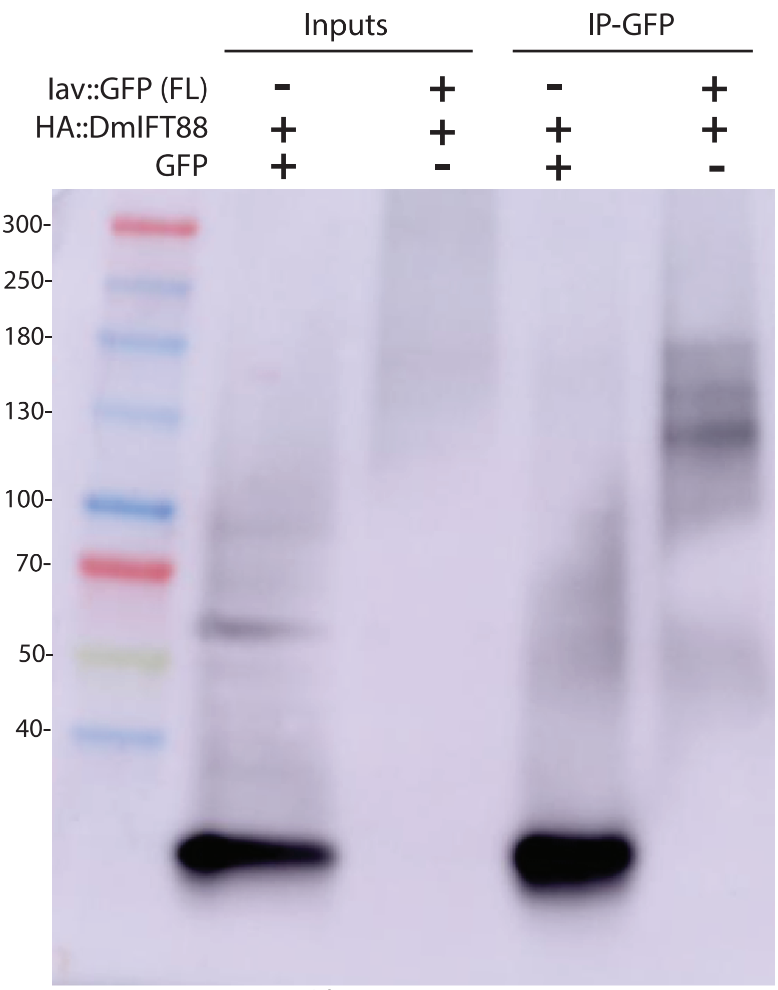

Blot used for lower Fig3A pannel (GFP)
and cut afterwards and reimaged for middle pannel

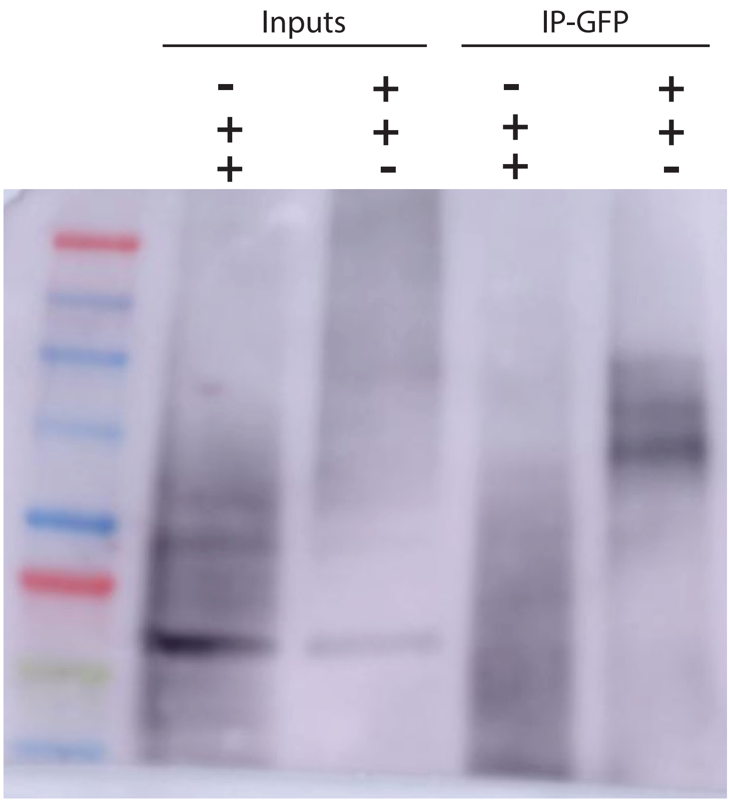

Upper portion of the left blot cut and reimaged with higher intensity,
used for Fig3A middle pannel (lav::GFP)

Anti-HA:

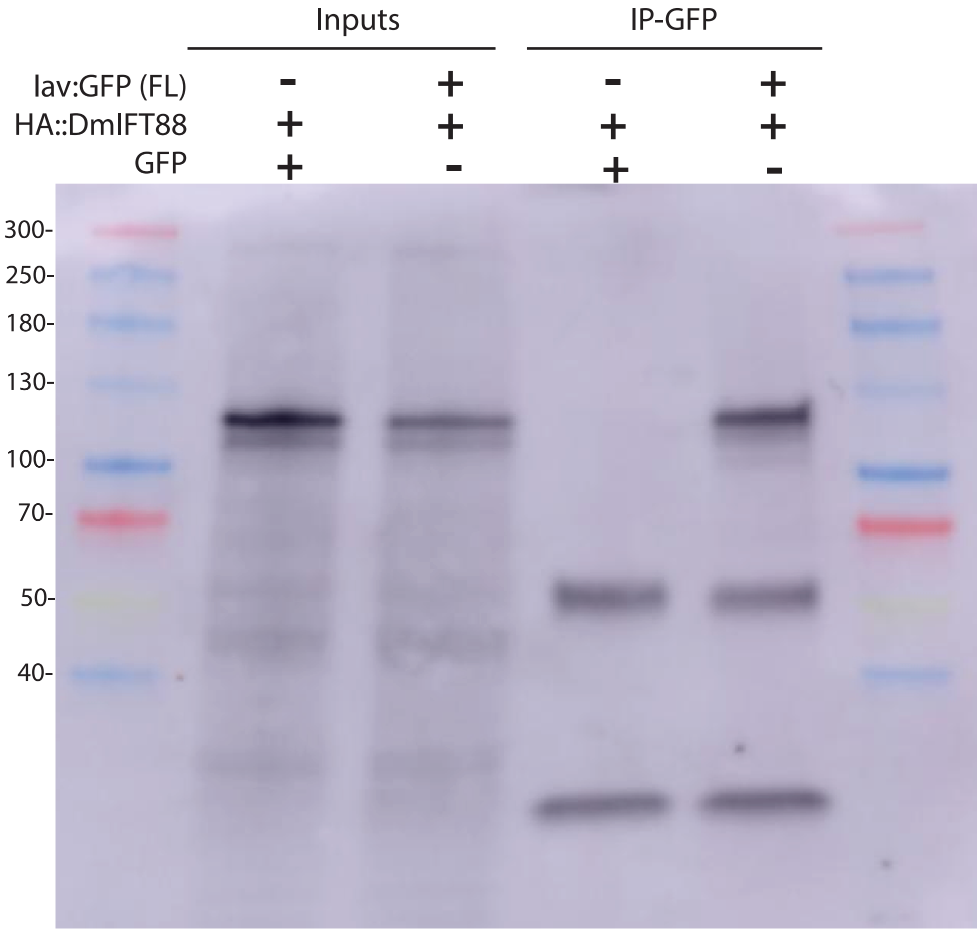

Blot used for Fig3A
upper pannel (DmIFT88)
