# Supplementary figures and images for: IFT88 maintains sensory function by localising signalling proteins along Drosophila cilia
Source: Life Sci Alliance. 2024 Feb 19;7(5):e202302289. doi: 10.26508/lsa.202302289 (PMC10876440; doi:10.26508/lsa.202302289)

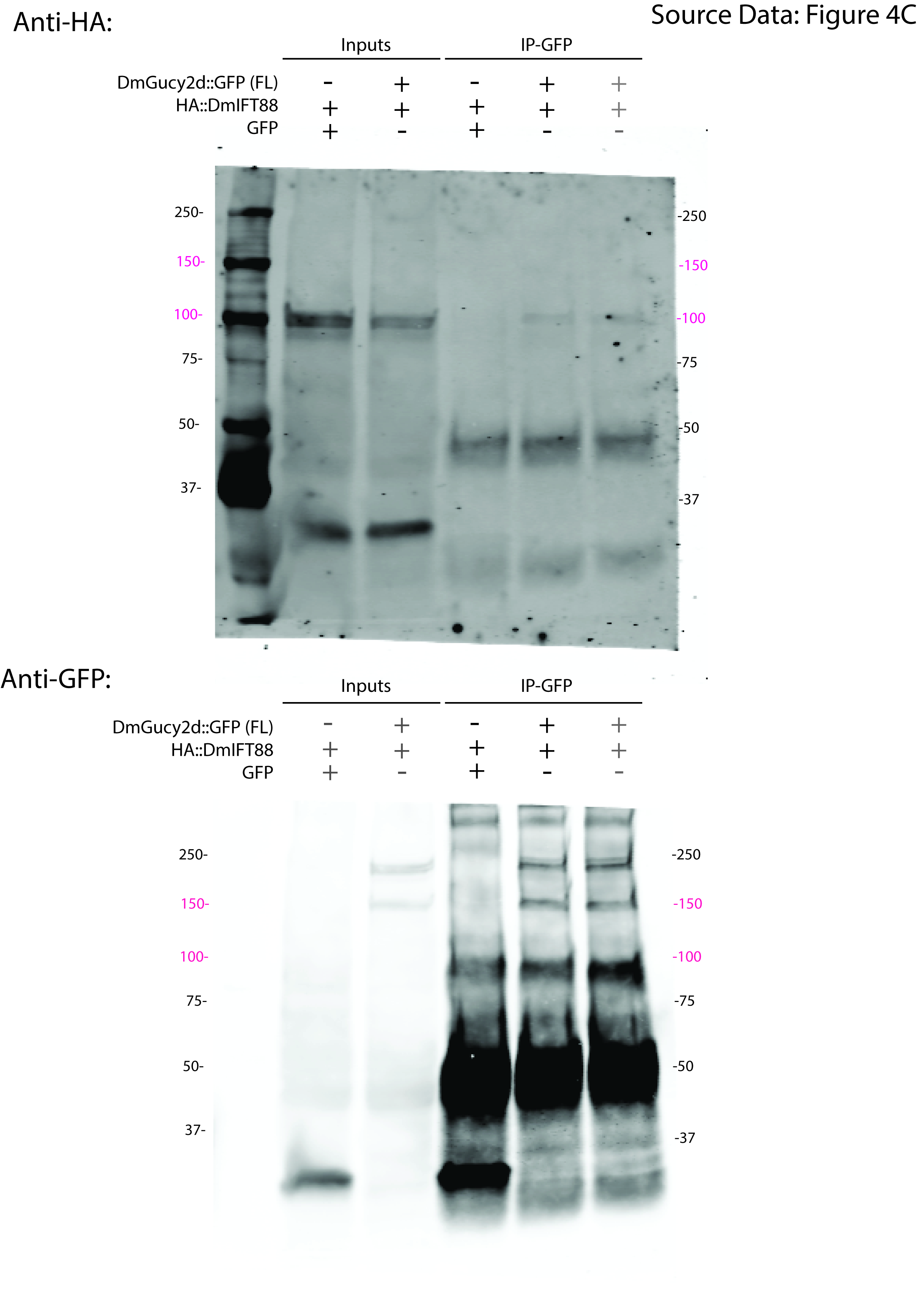

Supplement: Supplementary file 6 [file LSA-2023-02289_SdataF4.2.tif]

Anti-GFP:

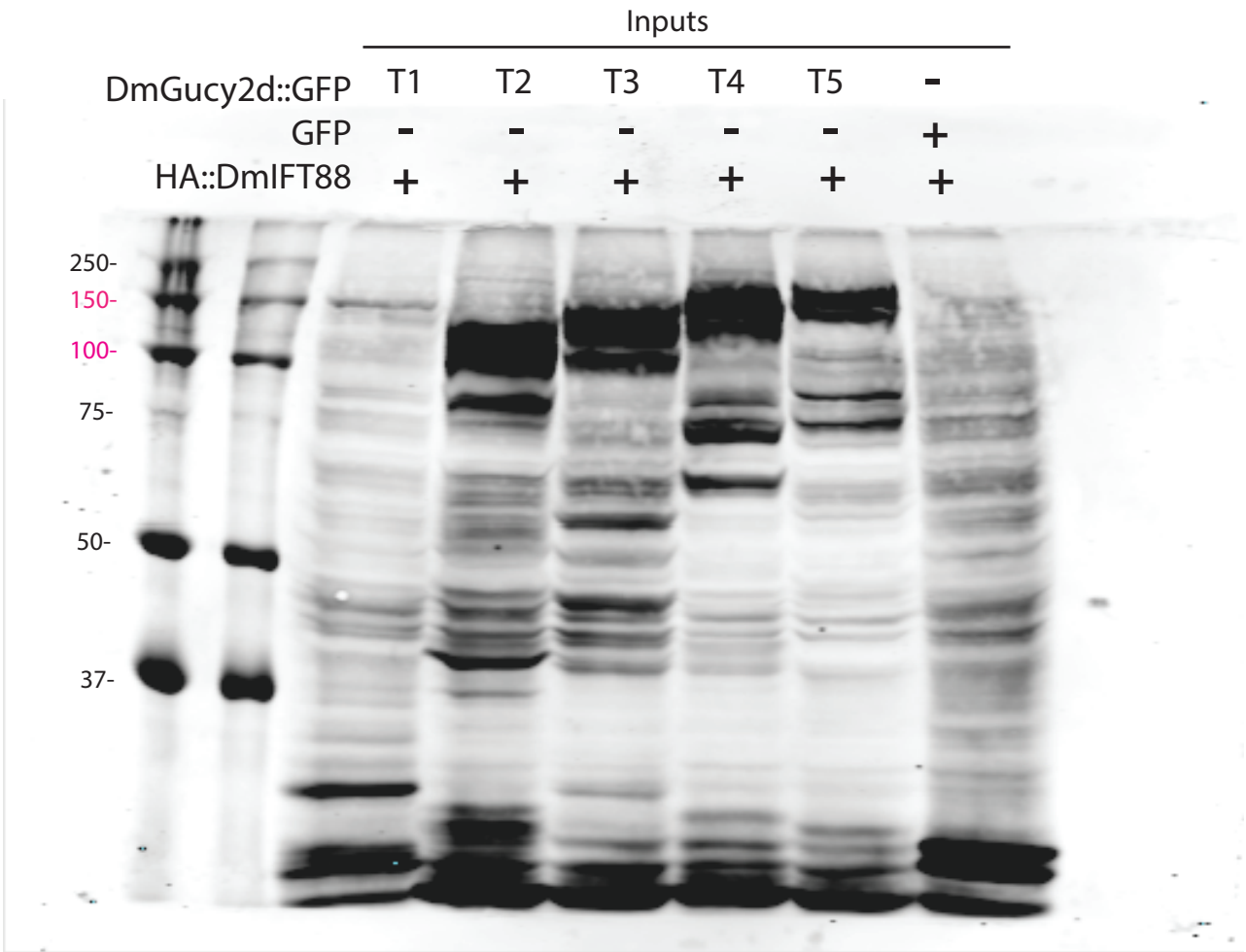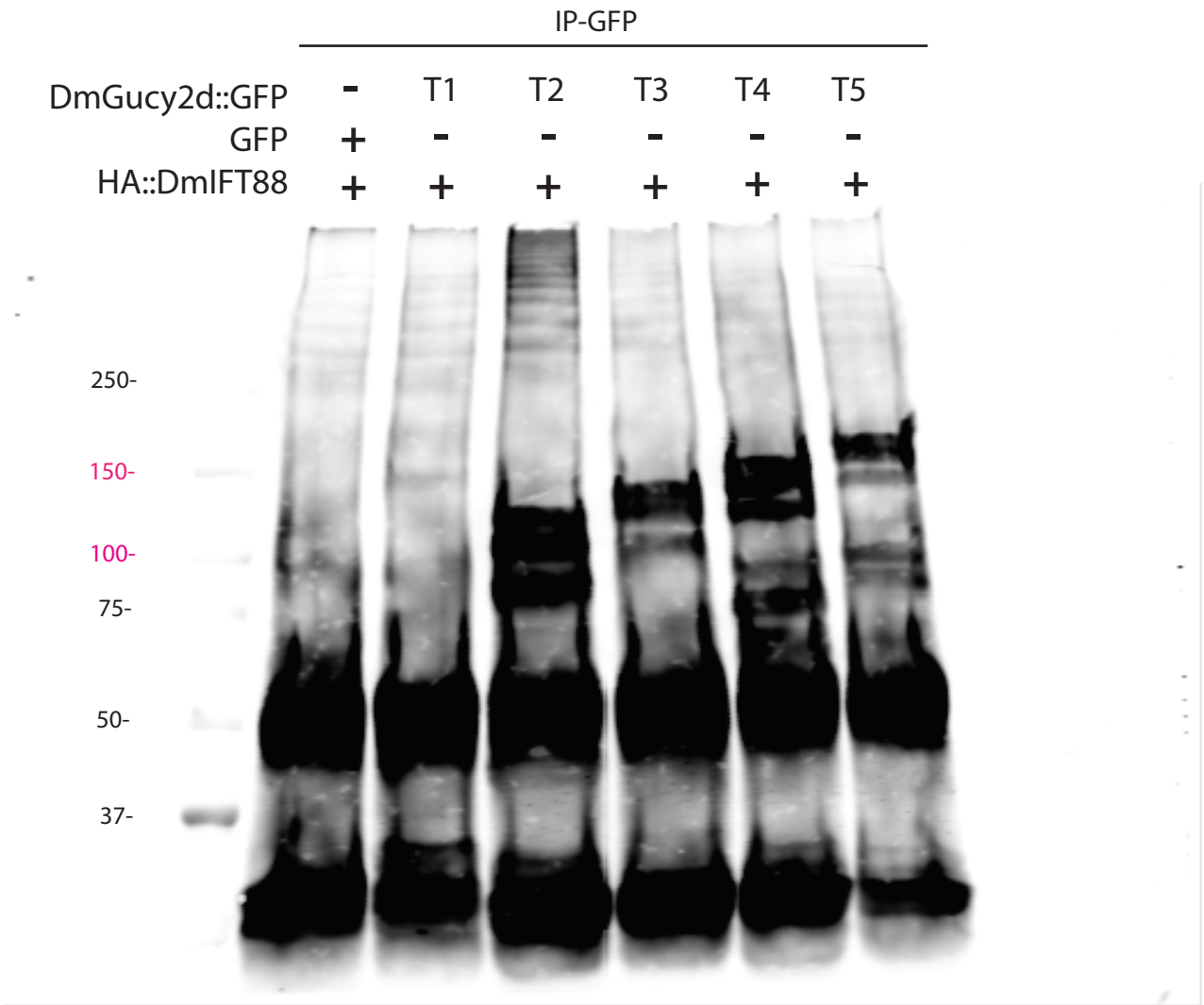

Supplement: Supplementary file 7 [file LSA-2023-02289_SdataF4.3.pdf]

Anti-HA:

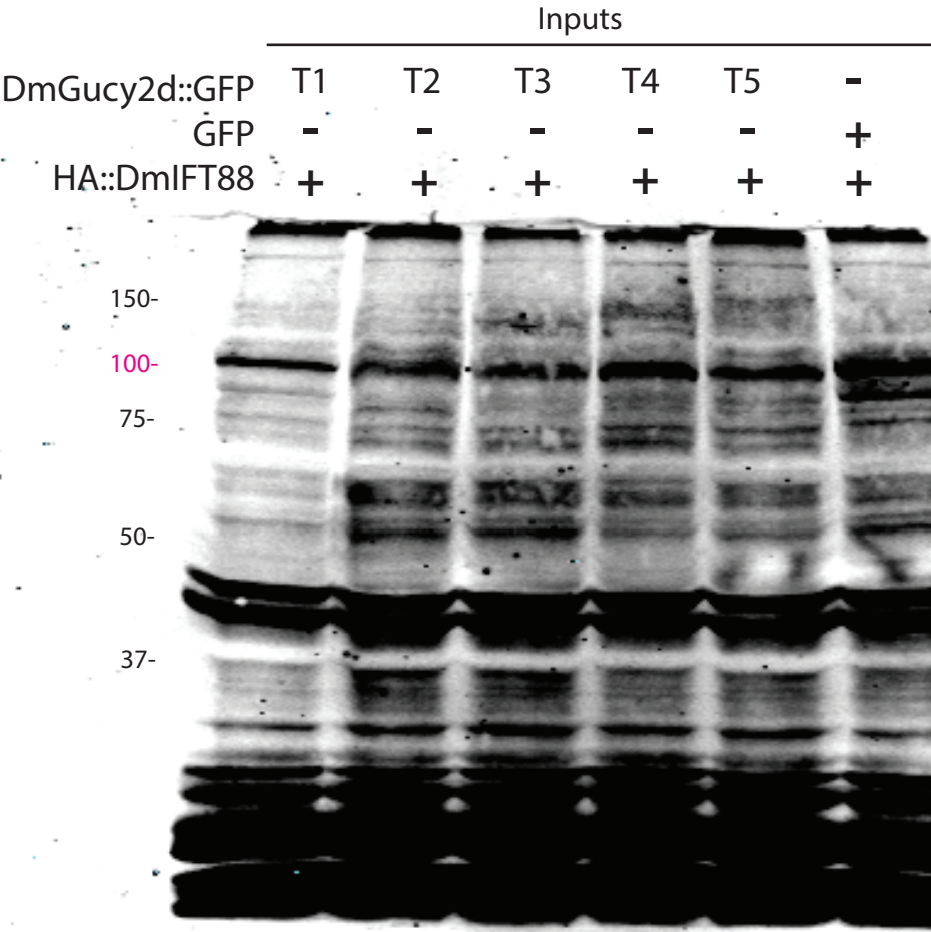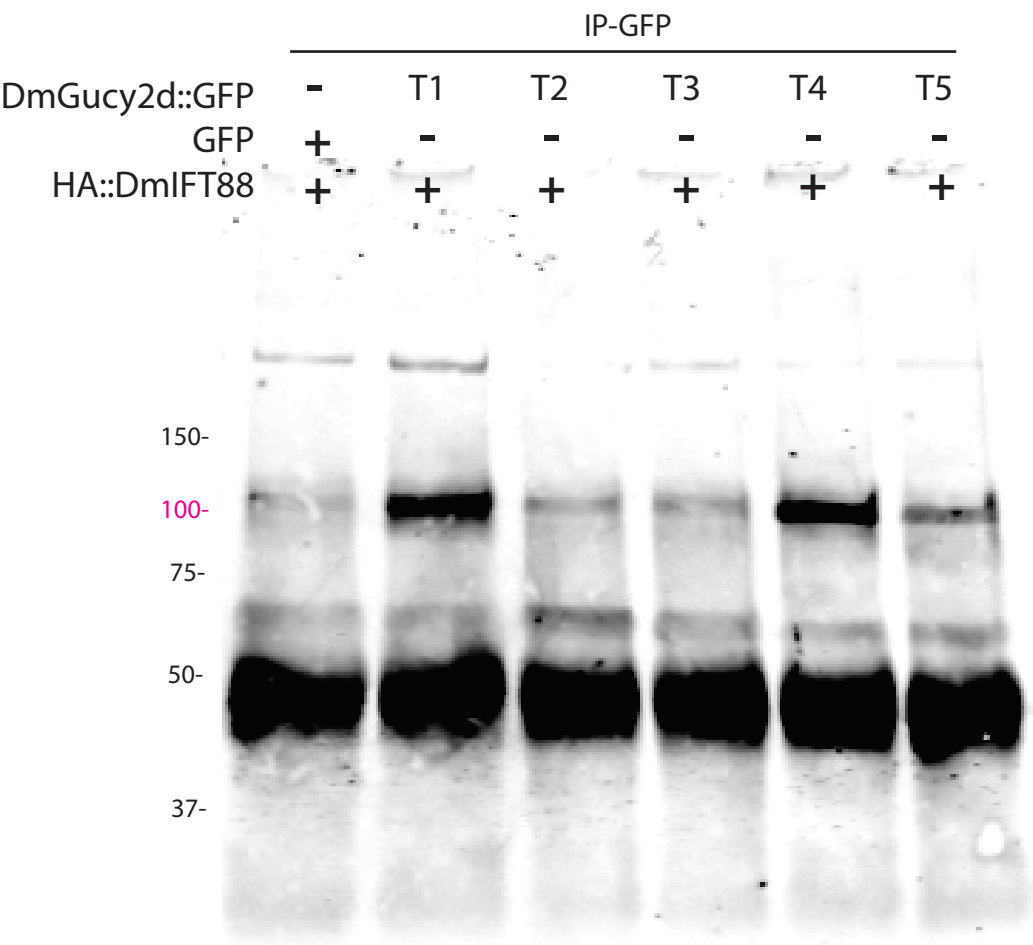

Supplement: Supplementary file 8 [file LSA-2023-02289_SdataF4.4.pdf]
