# Supplementary material for: IFT88 maintains sensory function by localising signalling proteins along Drosophila cilia
Source: Life Sci Alliance. 2024 Feb 19;7(5):e202302289. doi: 10.26508/lsa.202302289 (PMC10876440; doi:10.26508/lsa.202302289)
Supplement: Supplementary file 10 [file LSA-2023-02289_TableS1.docx]

| **Table S1** | | | |
| --- | --- | --- | --- |
|  | ***Chlamydomonas rheinhardii*** | ***Homo sapiens*** | ***Drosophila melanogaster*** |
| IFT-A  (6 members) | IFT43 | IFT43 | CG5780 |
|  | IFT121 | WDR35 | Oseg4 |
|  | IFT122/FAP | WDR10 | Oseg1 |
|  | IFT139 | THM1/TTC21B | *Not conserved* |
|  | IFT140 | WDTC2 | RempA/Oseg3 |
|  | IFT144 | WDR19 | Oseg6 |
| IFT-B  (16 members) | IFT20 | IFT20 | CG30441 |
|  | IFT22/FAP9 | RabL5 | *Not conserved* |
|  | IFT25/FAP232 | IFT25 | *Not conserved* |
|  | IFT27 | RabL4 | *Not conserved* |
|  | IFT38/FAP22 | Cluap | *Not conserved* |
|  | IFT46 | IFT46 | CG15161 |
|  | IFT52/Bld1 | IFT52 | Osm-6 |
|  | IFT54/FAP116 | TRAF3IP1 | CG3259 |
|  | IFT56/DYF-13 | TTC26 | *Not conserved* |
|  | IFT57 | Hippi | Che-13 |
|  | IFT70/FAP259 | TTC30A/B | *Not conserved* |
|  | IFT74 | IFT74 | *Not conserved* |
|  | IFT80 | IFT80 | Oseg5 |
|  | IFT81 | IFT81 | *Not conserved* |
|  | IFT88 | IFT88 | NompB |
|  | IFT172 | IFT172 | Oseg2 |
| BBSome  (10 members) | BBS1 | BBS1 | BBS1 |
|  | BBS2 | BBS2 | *Not conserved* |
|  | BBS3 | BBS3 | Arl6 |
|  | BBS4 | BBS4 | BBS4 |
|  | BBS5 | BBS5 | BBS5 |
|  | BBS6 | BBS6 | *Not conserved* |
|  | BBS7 | BBS7 | *Not conserved* |
|  | BBS8 | BBS8 | BBS8 |
|  | BBS9 | BBS9 | BBS9 |
|  | BBS18 | BBSIP10 | *Not conserved* |

**Table S1:** IFT and BBSome homologues in various species. *Drosophila* homologues were annotated according to https://flybase.org/.
