# Supplementary material for: IFT88 maintains sensory function by localising signalling proteins along Drosophila cilia
Source: Life Sci Alliance. 2024 Feb 19;7(5):e202302289. doi: 10.26508/lsa.202302289 (PMC10876440; doi:10.26508/lsa.202302289)
Supplement: Supplementary file 11 [file LSA-2023-02289_TableS2.docx]

| **Table S2** | | |
| --- | --- | --- |
| **Species** | **Abbreviations** | **Accession number** |
| ***Aedes aegypti*** | Ae | EAT40549 |
| ***Anopheles gambiae*** | Ag | XP_556988.3 |
| ***Danio rerio*** | Dr | XP_005167586 |
| ***Drosophila erecta*** | De | XP_001973983.2 |
| ***Drosophila melanogaster*** | Dm | NP_724347.3 |
| ***Drosophila simulans*** | Ds | EDX05743.1 |
| ***Gallus gallus*** | Gg | XP_015134793.1 |
| ***Homo sapiens*** | Hs | NP_001305422 |
| ***Mus musculus*** | Mm | NP_033402 |
| ***Tribolium castaneum*** | Tc | EFA00677 |
| ***Xenopus tropicalis*** | Xt | F6W448 |

**Table S2**: IFT88 protein sequences used for comparison.
