# Supplementary material for: IFT88 maintains sensory function by localising signalling proteins along Drosophila cilia
Source: Life Sci Alliance. 2024 Feb 19;7(5):e202302289. doi: 10.26508/lsa.202302289 (PMC10876440; doi:10.26508/lsa.202302289)
Supplement: Supplementary file 12 [file LSA-2023-02289_TableS3.docx]

| **Table S3** | | |
| --- | --- | --- |
| **Name** | **Species** | **Accession number** |
| **Hs_Gucy2d** | *Homo sapiens* | NP_000171 |
| **Mm_Gucy2e** | *Mus musculus* | NP_032218 |
| **Gg_retGC1** | *Gallus gallus* | AAC24500 |
| **Dr_retGC2** | *Danio rerio* | NP_001103165 |
| **Xt_retGC1** | *Xenopus tropicalis* | XP_002942678 |
| **Ms_GC-II** | *Manduca sexta* | AAN16469.1 |
| **Tc_retGC2** | *Tribolium castaneum* | KYB27701.1 |
| **Ag_AAEL007359-PA** | *Aedes aegypti* | XP_001658332.1 |
| **Ae_AGAP002233-PA** | *Anopheles gambiae* | XP_307952.5 |
| **Dm_CG10738** | *Drosophila melanogaster* | NP_729905.2 |
| **Dm_CG31183** | *Drosophila melanogaster* | NP_001287342.1 |
| **Dm_CG3216** | *Drosophila melanogaster* | NP_726013 |
| **Dm_CG34357** | *Drosophila melanogaster* | NP_001189166.1 |
| **Dm_Gyc32E** | *Drosophila melanogaster* | NP_001097148.1 |
| **Dm_Gyc76C** | *Drosophila melanogaster* | NP_001163473.1 |
| **Dm_Gyc89-Da** | *Drosophila melanogaster* | NP_001036718.1 |
| **Dm_Gyc89-Db** | *Drosophila melanogaster* | NP_650551.1 |
| **De_GG11511** | *Drosophila erecta* | XP_001978835 |
| **Ds_GD19673** | *Drosophila simulans* | XP_016033187 |

**Table S3**: Protein sequences used for bioinformatic analysis on guanylyl cyclases. Whenever several isoforms were available for the same gene, the longest protein sequence was chosen.
