# Supplementary material for: IFT88 maintains sensory function by localising signalling proteins along Drosophila cilia
Source: Life Sci Alliance. 2024 Feb 19;7(5):e202302289. doi: 10.26508/lsa.202302289 (PMC10876440; doi:10.26508/lsa.202302289)
Supplement: Supplementary file 14 [file LSA-2023-02289_TableS4.docx]

| **Table S4** | | | |
| --- | --- | --- | --- |
| **Species** | **Anterograde velocity** | **Retrograde velocity** | **References** |
| ***Mus musculus***  **(IMCD3 cells)** | 0.32 µm/s | 0.64 µm/s | ([Besschetnova et al., 2010](#_ENREF_8)) |
| ***Mus musculus***  **(IMCD3 cells)** | 1.19 µm/s | 1.0 µm/s | ([Ishikawa et al., 2014](#_ENREF_46)) |
| ***Mus musculus***  **(IMCD3 cells)** | 0.68 µm/s | 0.35 µm/s | ([Ye et al., 2013](#_ENREF_107)) |
| ***Mus musculus***  **(IMCD3 cells)** | 0.38 µm/s | 0.56 µm/s | ([Tran et al., 2008](#_ENREF_95)) |
| ***Mus musculus***  **(MEF)** | 1.09 µm/s | 1.06 µm/s | ([He et al., 2014](#_ENREF_42)) |
| ***Mus musculus***  ***(olfactory neurons)*** | 0.23 µm/s | 0.14 µm/s | ([Williams et al., 2014](#_ENREF_103)) |
| ***C. elegans*** | 0.71 µm/s (middle segment); 1.31 µm/s (distal segment) | 1.22 µm/s | ([Wei et al., 2012](#_ENREF_101)) |
| ***Drosophila melanogaster*** | 0.44 µm/s (proximal and distal segment) | 0.12 µm/s (proximal segment); 0.77 µm/s (outer segment) | ([Lee et al., 2018](#_ENREF_63)) |
| ***Drosophila melanogaster*** | 0.22 µm/s (proximal segment) | 1.2 µm/s (proximal segment) | This study |

**Table S4**: Overview of IFT88 velocities in mouse, *C. elegans* and *Drosophila* (including the results presented in this paper).
