# Supplementary material for: IFT88 maintains sensory function by localising signalling proteins along Drosophila cilia
Source: Life Sci Alliance. 2024 Feb 19;7(5):e202302289. doi: 10.26508/lsa.202302289 (PMC10876440; doi:10.26508/lsa.202302289)
Supplement: Supplementary file 16 [file LSA-2023-02289_TableS5.docx]

| **Table S5** | | |
| --- | --- | --- |
| **Name** | **Genotype** | **Source** |
| **Gal4*^Tubulin^*** | y[1] w[*]; P{w[+mC]=tubP-GAL4}LL7/ TM3, Sb[1] Ser[1] | BSC, # 5138 |
| **TubGal80^ts^** | w[*]; sna[Sco]/CyO;  P{w[+mC]=tubP-GAL80[ts]}7 | From Florence  Janody’s lab |
| **Gal4*^Chat19b^*** | w[*]; Chat-Gal4/CyO | ([Jana et al., 2011](#_ENREF_49)) |
| **Gal4 *^Chat19b^*, UAS-GFP** | w[*]; Chat-Gal4, UAS-GFP | ([Jana et al., 2021](#_ENREF_48)) |
| **Gal4*^Iav^*** | w[*];; Iav-Gal4 | BSC, # 52273 (a gift from Martin Göpfert’s lab) |
| **40xUAS-IVSmCD8::GFP** | w[*];; P{y[+t7.7] w[+mC]=40XUAS IVSmCD8::GFP} attP2 | BSC, #32195 |
| **UAS-mCD8::GFP** | w[*]; UAS-mCD8::GFP/CyO.Z;  MKRS/TM6B | Gift from Élio Sucena’s lab |
| **UAS-GFP::*Dm*IFT88** | W[1];;UAS-GFP::NompB/TM6B | This study (made using IGC Fly transgene facility) |
| **endo-GFP::*Dm*IFT88** | w[*]; endo-GFP::NompB | ([Han et al., 2003](#_ENREF_39)) (a gift from Daniel Eberl) |
| ***mCherry* RNAi** | y[1] sc[*] v[1]; P{y[+t7.7] v[+t1.8]=VALIUM20-mCherry}attP2 | BSC, # 35785 |
| ***DmIFT88* RNAi** | y[1] v[1]; P{y[+t7.7]  v[+t1.8]=TRiP.JF03080}attP2 | BSC, # 28665 |
| ***CG10738* RNAi 1** | y[1] v[1];; P{y[+t7.7]  v[+t1.8]=TRiP.HMS01814}attP2 | BSC, #38346 |
| ***CG10738* RNAi 2** | y[1] v[1];; P{y[+t7.7]  v[+t1.8]=TRiP.HM05067}attP2 | BSC, #28580 |
| ***CG31383* RNAi 1** | y[1] v[1];; P{y[+t7.7]  v[+t1.8]=TRiP.HM05092}attP2/TM3, Sb[1] | BSC, #28604 |
| ***CG31383* RNAi 2** | y[1] v[1]; P{y[+t7.7]  v[+t1.8]=TRiP.HMJ22232}attP40 | BSC, #58224 |
| ***CG3216* RNAi 1** | y[1] sc[*] v[1];; P{y[+t7.7]  v[+t1.8]=TRiP.HM05270}attP2/TM3, Sb[1] | BSC, #31877 |
| ***CG3216* RNAi 2** | y[1] v[1];; P{y[+t7.7]  v[+t1.8]=TRiP.HMC04174}attP2 | BSC, #55895 |
| ***DmGucy2d* RNAi 1 [GD29276]** | w1118;; UAS-IR[GD14677] | VDRC, #29276/GD |
| ***DmGucy2d* RNAi 2 [GD8469]** | w1118; UAS-IR[GD8469] | VDRC, #8469/GD |
| ***DmGucy2d* RNAi 3 [HM05010]** | y[1] v[1];; P{y[+t7.7]  v[+t1.8]=TRiP.HM05010}attP2 | BSC, #28524 |
| ***DmGucy2d* RNAi 4 [KK110863]** | w1118; UAS-IR[KK110863] | VDRC, #105185 |
| ***DmGucy2d* [NP0270]** | y[*] w[*];;  P{w[+mW.hs]=GawB}CG34357[NP0270] /TM6, P{w[-]=UAS-lacZ.UW23-1}UW23-1 | DGRC, #103574 |
| ***Gyc89-Da* RNAi** | y[1] sc[*] v[1];; P{y[+t7.7]  v[+t1.8]=TRiP.HM05246}attP2/TM3,Sb[1] | BSC, #30502 |
| ***Gyc89-Db* RNAi 1** | y[1] v[1];; P{y[+t7.7]  v[+t1.8]=TRiP.HM05207}attP2 | BSC, #29529 |
| ***Gyc89-Db* RNAi 2** | y[1] v[1]; P{y[+t7.7]  v[+t1.8]=TRiP.HMJ22088}attP40 | BSC, #58139 |
| **UAS-**  ***Dm*Gucy2d-T1::GFP** | w[*];; UAS- DmGucy2d -T1::GFP/TM6B | This study (made using IGC Fly transgene facility) |
| **UAS-**  ***Dm*Gucy2d::GFP** | w[*]; UAS-DmGucy2d::GFP/CyO | This study (made using BestGene facility) |
| **UAS-**  ***Dm*Gucy2d::GFP** | w[*];; UAS-DmGucy2d::GFP/TM3 | This study (made using BestGene facility) |

**Table S5**: Fly stocks used in this study. (BSC – Bloomington Stock Center, VDRC – Vienna Drosophila Resource Center, DGRC – Drosophila Genomics Resource Center)
