# Supplementary material for: IFT88 maintains sensory function by localising signalling proteins along Drosophila cilia
Source: Life Sci Alliance. 2024 Feb 19;7(5):e202302289. doi: 10.26508/lsa.202302289 (PMC10876440; doi:10.26508/lsa.202302289)
Supplement: Supplementary file 22 [file LSA-2023-02289_TableS6.docx]

| **Table S6** |
| --- |

**W640L**


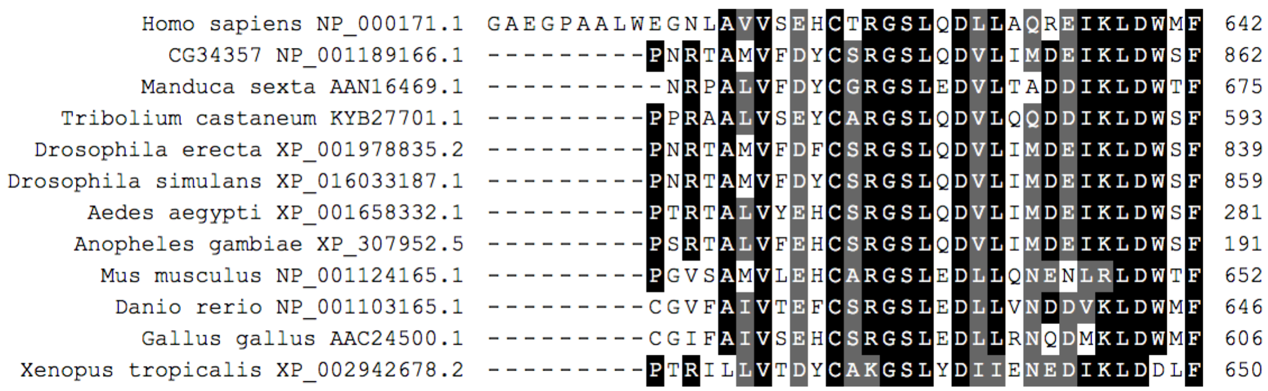


**A710V**


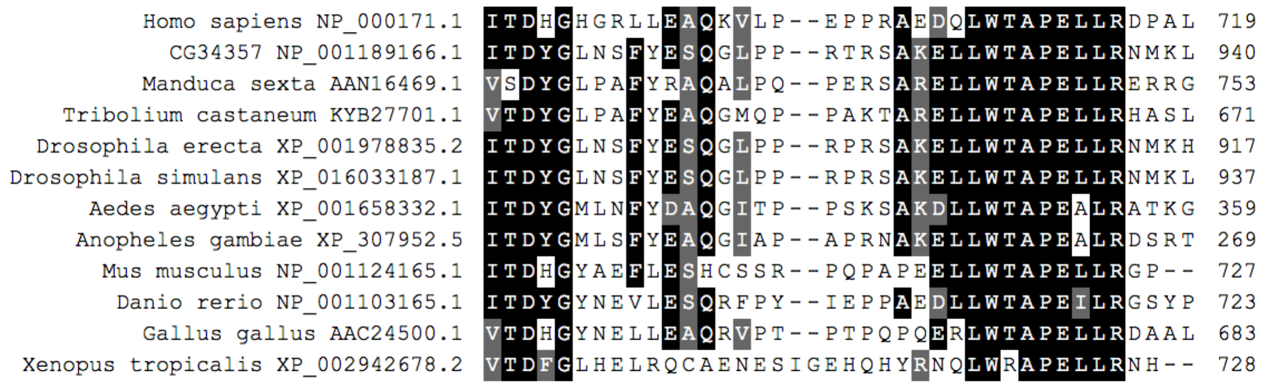


**I734A**


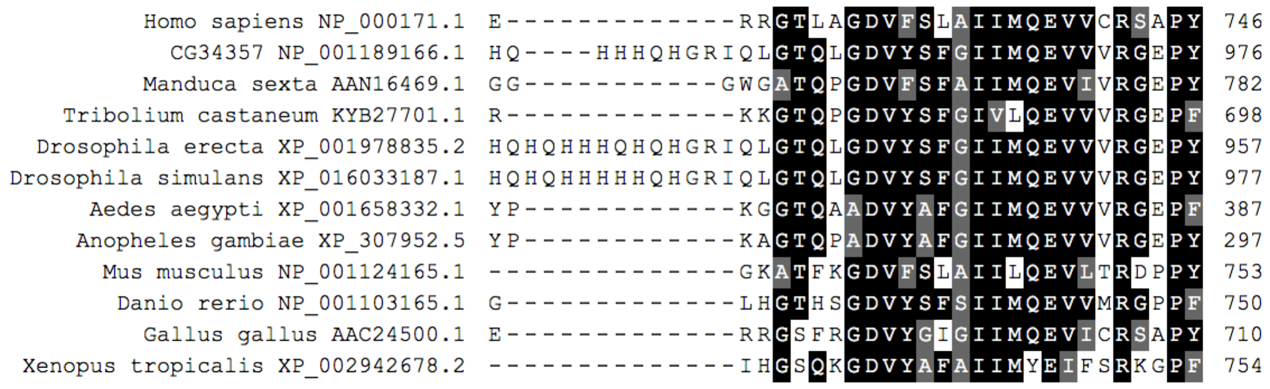


**R768W**


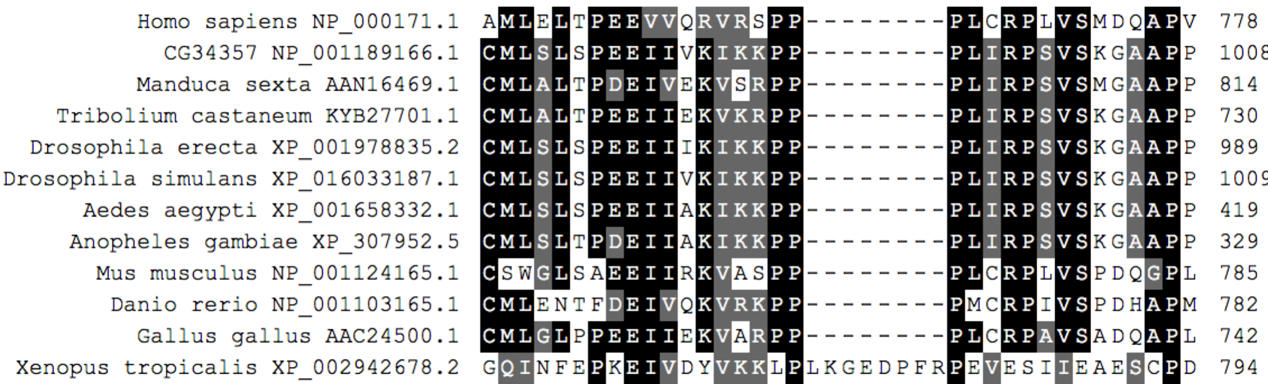


**P858S**


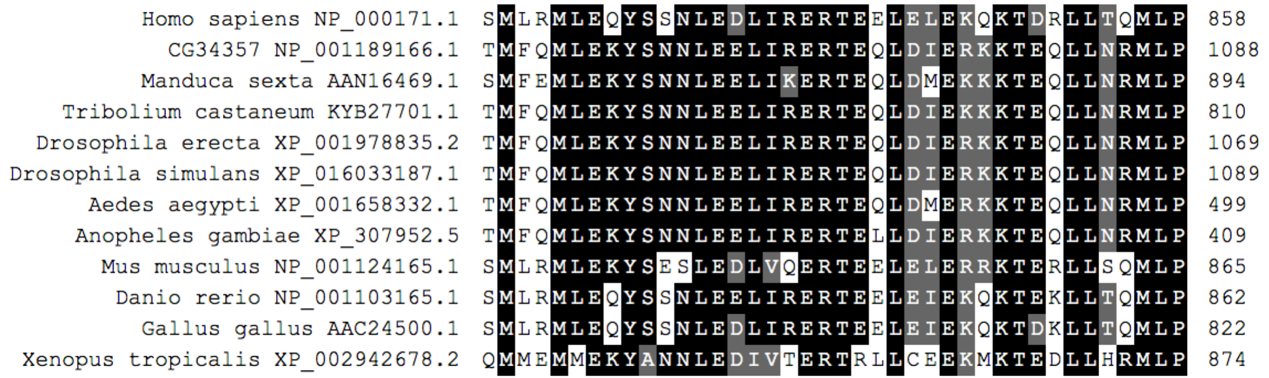


**L905P, I916T, G928Q**


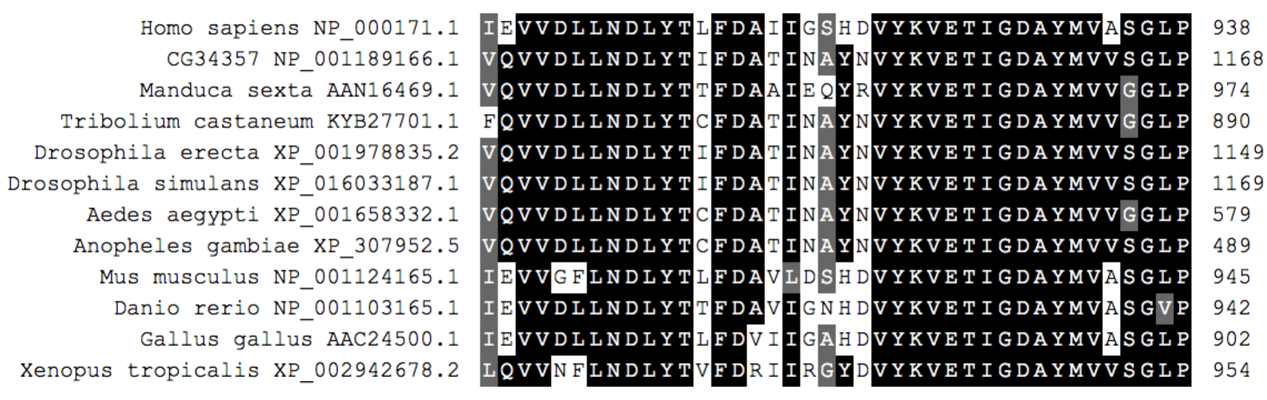


**L954P**


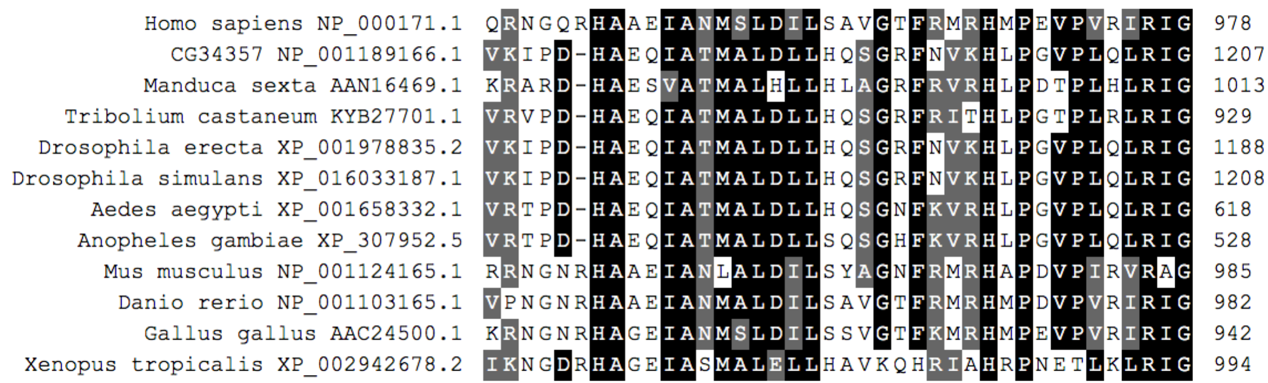


**H980L, A986Vfs*76**


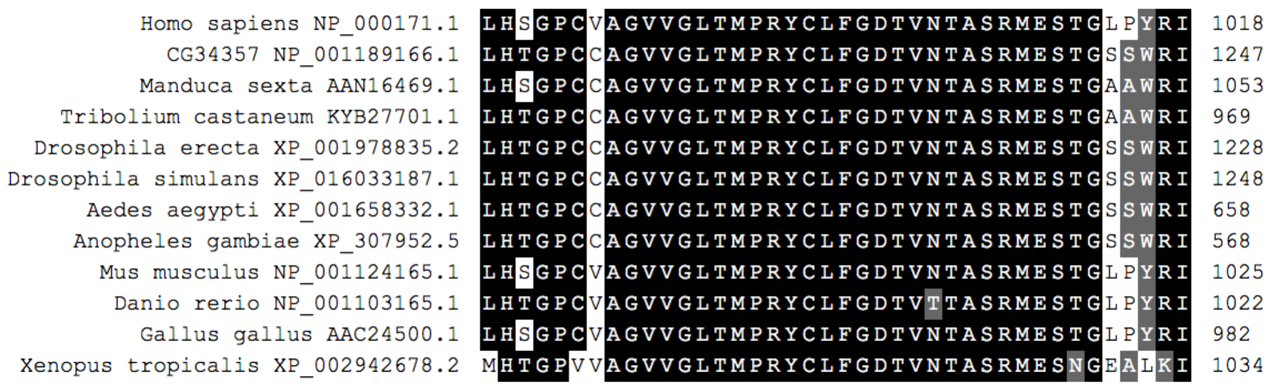


**H1019P**


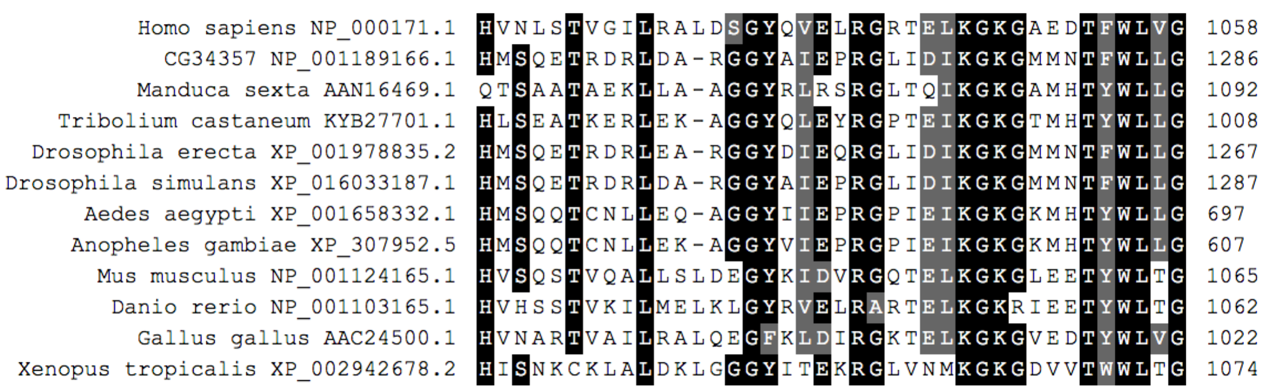


**P1069R**


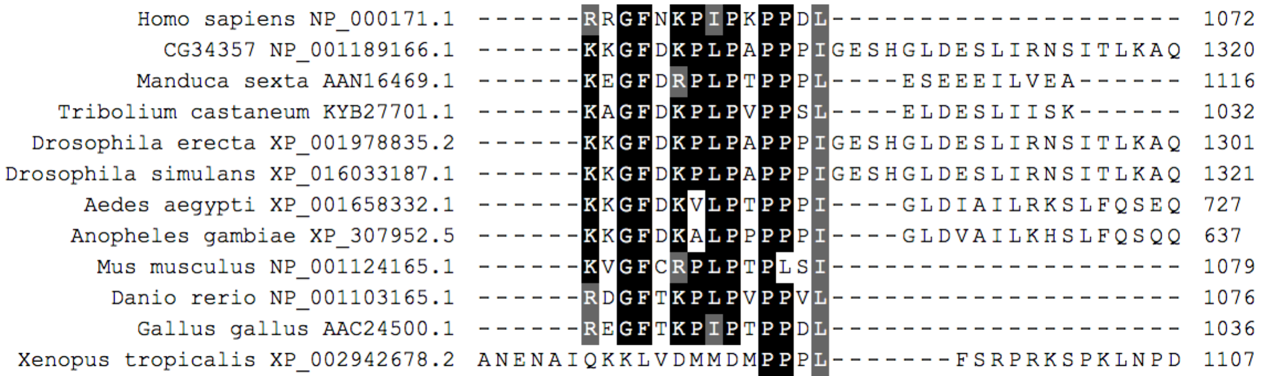


**Table S6**: Screenshots of alignments of selected sequences of intracellular domains of Gucy2d from diverse species visualised with “Multiple Alignment Show”. Sequences were aligned using **MU**ltiple **S**equence **C**omparison by **L**og- **E**xpectation (MUSCLE) ([Madeira et al., 2022](#_ENREF_69)) . In addition to the six species shown in Supplemental Figure 5A, six insect species were included in the analysis to avoid a vertebrate bias in the multiple sequence alignment (Table S3). Note that the residues that are mutated in human LCA patients and are evolutionarily conserved are marked with red boxes ([de Castro-Miro et al., 2014](#_ENREF_23); [Feng et al., 2020](#_ENREF_29); [Jacobson et al., 2013](#_ENREF_47); [Li et al., 2011](#_ENREF_65); [Liu et al., 2020](#_ENREF_68); [Salehi Chaleshtori et al., 2020](#_ENREF_86); [Tucker et al., 2004](#_ENREF_97); [Zagel and Koch, 2014](#_ENREF_109)).
