# Supplementary material for: IFT88 maintains sensory function by localising signalling proteins along Drosophila cilia
Source: Life Sci Alliance. 2024 Feb 19;7(5):e202302289. doi: 10.26508/lsa.202302289 (PMC10876440; doi:10.26508/lsa.202302289)
Supplement: Supplementary file 23 [file LSA-2023-02289_TableS7.docx]

| **Table S7** | | | |
| --- | --- | --- | --- |
| **Antibody** | **Dilution** | **Species** | **Source** |
| **Primary antibodies** | | | |
| anti-GFP | 1:1000 | rabbit | Invitrogen, USA |
| anti-GFP | 1:1000 | chicken | Aves, USA |
| anti-GFP | 1:1000 | rabbit | Roche, Germany |
| anti-GFP | 1:1000 | rabbit | Abcam, UK |
| anti-HA | 1:1000 | mouse | Biolegend, UK |
| anti-PLP | 1:1000 | chicken | ([Fu and Glover, 2012](#_ENREF_32)) |
| anti-Glutamylated tubulin GT335 | 1:500 | mouse | ([Wolff et al., 1992](#_ENREF_105)) |
| anti-NompC | 1:200 | rabbit | ([Cheng et al., 2010](#_ENREF_20)) |
| anti-Iav | 1:500 | rat | ([Gong et al., 2004](#_ENREF_33)) |
| anti-acetylated tubulin | 1:500 | mouse | Sigma, USA |
| **Secondary antibodies** | | | |
| anti-chicken FITC | 1:500 | donkey | Jackson Labs, USA |
| anti-rat Rhodamine | 1:500 | donkey | Jackson Labs, USA |
| anti-mouse IRDye 680 | 1:10000 | goat | LI-COR, USA |
| anti-mouse IRDye 800 | 1:10000 | goat | LI-COR, USA |
| anti-rabbit IRDye 800 | 1:10000 | goat | LI-COR, USA |
| anti-rabbit Alexa647 | 1:10000 | donkey | Jackson Labs, USA |
| anti-rabbit Alexa488 | 1:500 | goat | Molecular Probes, USA |
| anti-rabbit Alexa647 | 1:500 | donkey | Jackson Labs, USA |
| anti-chicken Rhodamine | 1:500 | donkey | Jackson Labs, USA |
| anti-rat Rhodamine | 1:500 | goat | Jackson Labs, USA |
| anti-mouse Alexa488 | 1:500 | goat | Molecular Probes, USA |
| anti-mouse Alexa647 | 1:500 | goat | Life technologies, USA |

**Table S7**: Details of the antibodies used in this paper.
