# Supplementary material for: IFT88 maintains sensory function by localising signalling proteins along Drosophila cilia
Source: Life Sci Alliance. 2024 Feb 19;7(5):e202302289. doi: 10.26508/lsa.202302289 (PMC10876440; doi:10.26508/lsa.202302289)
Supplement: Supplementary file 24 [file LSA-2023-02289_TableS8.docx]

| **Table S8** | | | |
| --- | --- | --- | --- |
| **Name** | **Purpose** | **Sequence** | **T_a_** |
| **Dm*IFT88*_FL_gateway_fwd** | Gateway cloning  *DmIFT88* | GGGACAAGTTTGTACA  AAAAAGCAGGCTTCAT  GACTTCTCAAATAACT  GCTAACGGAACGC | 52°C |
| **Dm *IFT88*_FLS_gateway_rev** | Gateway cloning *DmIFT88* (with Stop codon) | GGGGACCACTTTGTAC AAGAAAGCTGGGTCTC  AAATAGGCAATAAGCT  TTCGGG | 52°C |
| **Dm *IFT88*_seq_fwd** | For sequencing *DmIFT88* coding sequence | GCAGCCACAGTGAAACATCG | -- |
| **Dm *IFT88*_seq_rev** | For sequencing *DmIFT88* coding sequence | AACTCAGGTTGGTCAGAGCG | -- |
| **Dm *IFT88*_seq_rev2** | For sequencing *DmIFT88* coding sequence | AGACGGGTGTATTCCGATGC | -- |
| **Dm *IFT88*-RD_RT-PCR_fwd** | Quantify mRNA expression of  *DmIFT88*-RD isoform | CCCCTACACGTCCATTCTGC | 54°C |
| **Dm *IFT88*-RD_RT-PCR_rev** | Quantify mRNA expression of  *DmIFT88*-RD isoform | CCCCTACACGTCCATTCTGC | 54°C |
| **Dm *IFT88*_RC_RT-PCR_fwd** | Quantify mRNA expression of  *DmIFT88*-RC isoform | CGCTGGAATTAGGGGACCTG | 54°C |
| **Dm *IFT88*-RC_RT-PCR_rev2** | Quantify mRNA expression of  *DmIFT88*-RC isoform | AGGATCAAACTGCAGAAATCG | 54°C |
| ***elF*-1A_RT-PCR_fwd** | Quantify mRNA expression of *elF1A* (house keeping gene) | GATATACTGGTTCCCCGCGA | 54°C |
| ***elF*-1A_RT-PCR_rev** | Quantify mRNA expression of *elF1A* (house keeping gene) | GGCTTGTTGGCGACCAATTTT | 54°C |
| ***Su(Tpl)*_RT-PCR_fwd** | Quantify mRNA expression of *Su(Tpl)* (house keeping gene) | AGCCACAAATCCATGCAGAG | 54°C |
| ***Su(Tpl)*_RT-PCR_rev** | Quantify mRNA expression of *Su(Tpl)* (house keeping gene) | TGGACGTTGACTTCTTGTTGT | 54°C |
| **TBP_RT-PCR_fwd** | Quantify mRNA expression of *TBP* (house keeping gene) | TTATGCGAATCCGAGAGCCC | 54°C |
| **TBP_RT-PCR_rev** | Quantify mRNA expression of *TBP* (house keeping gene) | GTCGAGGAACTTTGCAGGGA | 54°C |
| **Dm*Gucy2d* (*CG34356*)_5UTR_fwd** | Outer primer pair for nested PCR strategy to amplify FL-*DmGucy2d* coding sequence | GGCAATGTTGGAAGGCACTG | 52°C |
| **Dm*Gucy2d*_3UTR_rev** | See above | CCCATGTCGTCAGCCATCTAA |  |
| **Dm*Gucy2d*_gateway_FLfwd** | Gateway cloning of FL *DmGucy2d* | GGGGACAAGTTTGTACAAAAAAGCAGGCTTCATGAAGTTAACAACCTGTCAAATTGCTAAAG | 53°C |
| **Dm*Gucy2d*_gateway_FLrev** | Gateway cloning of FL *DmGucy2d* | GGGGACCACTTTGTACAAGAAAGCTGGGTCTCTGCTCAGCAGTTGCTCTGCATTAAGGG | 53°C |
| **Dm*Gucy2d*_trunc_fwd** | Gateway cloning of  T1-*DmGucy2d* | GGGGACAAGTTTGTACAAAAAAGCAGGCTTCATGCGTAAGCGTCTCTCCAAGGG | 53°C |
| **Dm*Gucy2d*_trunc_rev1** | Gateway cloning of  T2-*DmGucy2d* | GGGGACCACTTTGTACAAGAAAGCTGGGTCGTTCGCCGTGGTTGTGATG | 53°C |
| **Dm*Gucy2d*_trunc_rev2** | Gateway cloning of  T3-*DmGucy2d* | GGGGACCACTTTGTACAAGAAAGCTGGGTCTCTGGTTCTTGGCGGAAGTC | 53°C |
| **Dm*Gucy2d*_trunc_rev3** | Gateway cloning of  T4-*DmGucy2d* | GGGGACCACTTTGTACAAGAAAGCTGGGTCAAATTCTTCCGGGTCGACGG | 53°C |
| **Dm*Gucy2d*_trunc_rev4** | Gateway cloning of  T5-*DmGucy2d* | GGGGACCACTTTGTACAAGAAAGCTGGGTCAGATTCTCCAATAGGCGGCG | 53°C |
| **Dm*Gucy2d*_seq_fwd1** | To confirm PCR products through sequencing | TGGCAGGACAATACGTGACC | -- |
| **Dm*Gucy2d*_seq_fwd2** | To confirm PCR products through sequencing | GGACTGGTCGTTTCGGCTAA | -- |
| **Dm*Gucy2d*_seq_fwd3** | To confirm PCR products through sequencing | TCCTGGCGCATCCATATGTC | -- |
| **Dm*Gucy2d*_seq_rev1** | To confirm PCR products through sequencing | GAACCCATGCTGAGTGTCCA | -- |
| **Dm*Gucy2d*_seq_rev2** | To confirm PCR products through sequencing | TTTCTCGAGGTCAAGGCACC | -- |
| **Dm*Gucy2d*_seq_rev3** | To confirm PCR products through sequencing | GGTGGAATTGGAGTCCAGGG | -- |

**Table S8**: Primers used for the experiments in this paper and the annealing temperatures (T_a_) used. The OligoCalc tool was used for prediction (Kibbe, 2007); extension times were chosen according to the length of expected product and the properties of the respective polymerase according to the manufacturer.
